# Transcranial Electrical Stimulation targeting limbic cortex increases the duration of human deep sleep

**DOI:** 10.1016/j.sleep.2021.03.001

**Published:** 2021-03-08

**Authors:** Evan Hathaway, Kyle Morgan, Megan Carson, Roma Shusterman, Mariano Fernandez-Corazza, Phan Luu, Don M. Tucker

**Affiliations:** a Brain Electrophysiology Laboratory, Co., Eugene, OR, USA; b University of Oregon Department of Psychology, Eugene, OR, USA; c LEICI Instituto de Investigaciones en Electrónica, Control y Procesamiento de Señales, Universidad Nacional de La Plata, CONICET, Argentina

**Keywords:** EEG, Sleep, Memory, Slow oscillations, Deep sleep, Slow wave sleep

## Abstract

**Background:**

Researchers have proposed that impaired sleep may be a causal link in the progression from Mild Cognitive Impairment (MCI) to Alzheimer’s Disease (AD). Several recent findings suggest that enhancing deep sleep (N3) may improve neurological health in persons with MCI, and buffer the risk for AD. Specifically, Transcranial Electrical Stimulation (TES) of frontal brain areas, the inferred source of the Slow Oscillations (SOs) of N3 sleep, can extend N3 sleep duration and improve declarative memory for recently learned information. Recent work in our laboratory using dense array Electroencephalography (dEEG) localized the sources of SOs to anterior limbic sites – suggesting that targeting these sites with TES may be more effective for enhancing N3.

**Methods:**

For the present study, we recruited 13 healthy adults (*M* = 42 years) to participate in three all-night sleep EEG recordings where they received low level (0.5 mA) TES designed to target anterior limbic areas and a sham stimulation (placebo). We used a convolutional neural network, trained and tested on professionally scored EEG sleep staging, to predict sleep stages for each recording.

**Results:**

When compared to the sham session, limbic-targeted TES significantly increased the duration of N3 sleep. TES also significantly increased spectral power in the 0.5–1 Hz frequency band (relative to pre-TES epochs) in left temporoparietal and left occipital scalp regions compared to sham.

**Conclusion:**

These results suggest that even low-level TES, when specifically targeting anterior limbic sites, can increase deep (N3) sleep and thereby contribute to healthy sleep quality.

## 1. Introduction

Impaired sleep may be a causal factor in the progression from healthy aging to Mild Cognitive Impairment (MCI) and eventually to Alzheimer’s Disease (AD) [1]. Not only does sleep restriction lead to accumulation of beta amyloid proteins, but the onset of sleep impairment is typically observed well before the clinical presentation of AD [2]. A physiological mechanism for the neurotoxic effects of sleep impairment is provided by evidence that the brain’s glymphatic (glial-lymph) removal of metabolic toxins may be limited to the period of sleep [3]. Recent evidence that slow wave sleep (Non-REM Stage 3 or N3) is specifically required for glymphatic clearance of neurotoxins in humans further supports the causal role of sleep fragmentation in the pathophysiology of mental decline in AD [4]. The important implication of these findings is that improving deep sleep could prevent the silent, chronic neurotoxicity that causes dementia.

**Fig. 1.**
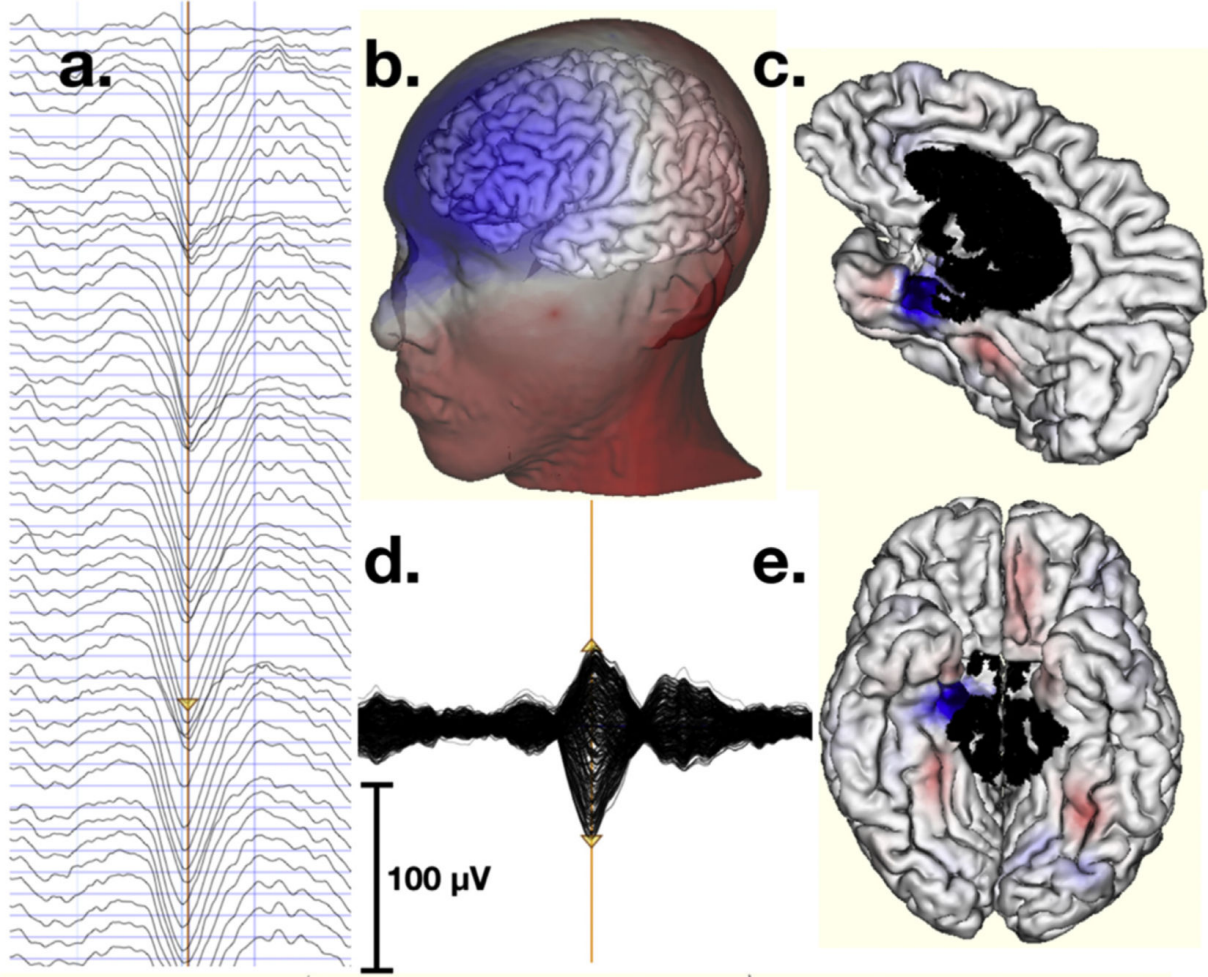
Targeting of SO sources with TES begins with electrical source localization of SOs. **a.** SO in the frontal EEG channels 1–43. Positive is up and vertical blue lines are 1-sec marks. **b.** Head surface topography at the negative peak of the SO marked by the brown vertical synch line in a. The SO has a large negative deflection in frontal channels coincident with a posterior positive field. **c. & e.** Source localization of the negative peak of the SO to the individual’s cortical surface. The main source of the SO is a tight source-sink inversion over the right anterior parahippocampal gyrus. **d.** Butterfly plot (all channels) of the SO.

The defining EEG features of deep sleep are the large (>75 μV) Slow Oscillations (SOs). Massimini et al. (2004) reported that SOs reflect traveling waves that originate in frontal regions and propagate over widespread areas of neocortex [5]. Although additional studies using dense array EEG to measure SOs have been reported more recently [6,7], none have directly challenged the conclusion that SOs reflect global traveling waves with frontal onset. In examining dense array EEG recordings of human N3 sleep in our own lab, we observed that the typical SO pattern of a frontal-negative potential field was invariably accompanied by a simultaneous positive potential field over posterior regions. The dipolar inversion of the field suggests the neural source of the typical SO is then located in the anterior temporal lobe (see Fig. 1 b). In twelve normal young adults, we conducted detailed electrical source analysis of dense array sleep EEG, finding that the great majority of SOs in young adults are generated by anterior ventral limbic cortex, including the parahippocampal gyrus of medial temporal lobes and the caudal regions of orbitofrontal cortex (Morgan et al., unpublished work).

Although previous studies have demonstrated successful synchronization of SOs with slow TES pulses [5,8,9], the stimulation in those studies used electrodes in dorsolateral frontal areas (F3, F4 or F7, F8 versus contralateral mastoids), consistent with the assumption that human SOs emanate from frontal neocortex. In contrast, once we knew to target the anterior limbic sources of SOs, straightforward computational simulation showed that electrodes at frontopolar and inferior frontal head sites (versus posterior return electrodes) would be most effective in targeting the limbic sources of SOs. In the present study, we hypothesized that Transcranial Electrical Stimulation (TES) could be applied to frontopolar and inferior frontal head sites in order to synchronize the limbic sources of SOs specifically and thereby enhance the duration of N3 sleep. Furthermore, based on our computational modeling with this more optimal targeting of the limbic sites, we hypothesized that we could use lower TES current levels (0.5 mA versus 1 or 2 mA in previous studies) that would be unlikely to disrupt sleep and that may still be successful in synchronizing SOs to enhance the adaptive neurophysiology of deep sleep.

## 2. Materials and methods

### 2.1. Subjects

Thirteen healthy adults (6 women, 7 men) ranging between 20 and 67 years old (*M* = 42) participated in the study. Subjects were screened to exclude those with a history of seizures, epilepsy, brain trauma or injury, insomnia, and sleep apnea, or those using medications that may affect the EEG. Two subjects were omitted from analysis for poor data quality and one subject was omitted for failing to initially disclose the consumption of an excluded medication, resulting in 10 subjects being retained for analysis. Informed consent was obtained from all subjects prior to their participation in the experiment. The plan was for a representative sample (*N* = 60) including younger and older adults, but the study was halted in March 2020 after collecting 13 participants because of COVID-19 restrictions.

### 2.2. Experimental design

All experimental protocols were approved by the Oregon Research Institute (ORI) Institutional Review Board. All study sessions were conducted in the Brain Electrophysiology Laboratory (BEL) located in Eugene, OR, USA. The experiment consisted of three overnight sessions lasting approximately 8 hr; each beginning at 11:00 PM and ending around 7:00 AM the next morning. The first session was an adaptation night, consisting of a full-night EEG recording. During the second and third sessions, subjects received either TES or a sham (placebo) protocol in addition to EEG monitoring. The second and third sessions were spaced one week apart to allow any effects of TES or sham to diminish. The order of sham and TES were counterbalanced across subjects to control for order effects. Subjects were blinded to the condition being delivered during nights 2 and 3. Experimenters were also blinded to the experimental condition until they opened an email that specified the treatment condition for that night after the subject was asleep.

### 2.3. EEG recording and Transcranial Electrical Stimulation

**Fig. 2.**
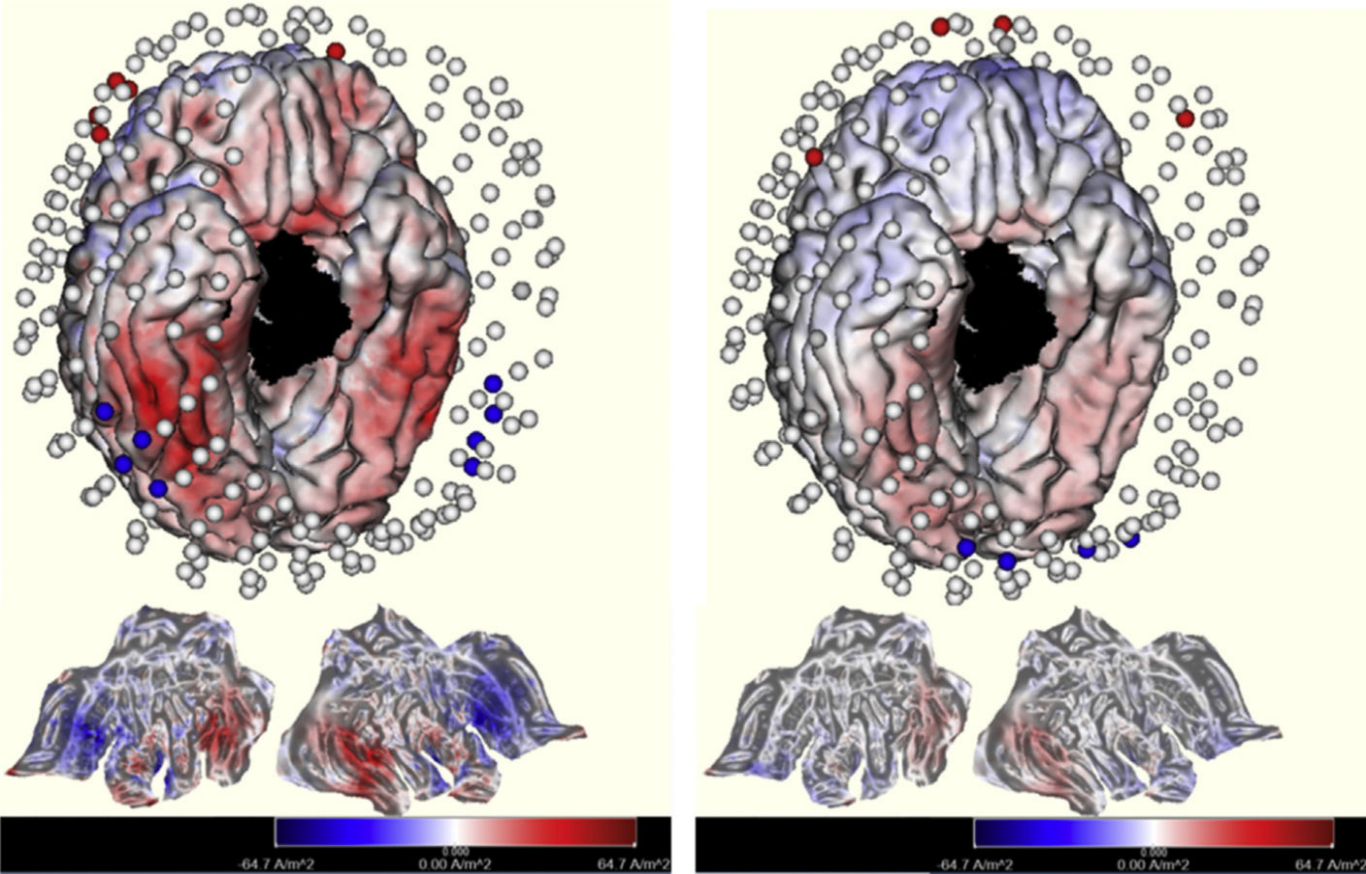
Targeting of SO sources with the dorsolateral frontal vs mastoid sites used in Marshall et al. (2006) (**left**) compared to the present frontopolar (near Fp1, Fp2) and inferolateral sites (near F9 and F10 of the International Ten-Twenty System) (**right**). Each of these cortical current delivery simulations is calibrated by the magnitude of impressed current used in each study (~1 mA in Marshall and ~0.5 mA in the present study). The anterior electrodes are shown as sources (red) and the posterior as sinks (blue). For the Marshall et al. positions we used four adjacent electrodes to account for the likely positioning of the F3, F4 sources, and used bilateral mastoid electrodes for sinks. At bottom are cortical flat maps, created by unfolding the 3D cortical surface to a 2D representation.

EEG was recorded and TES delivered with a 256-channel Geodesic Sensor Net (Elefix conductive paste) and a Net Amps 400 GTEN Amplifier (EGI/Philips Neuro, Eugene, OR). EEG was sampled at a rate of 1000 Hz and referenced to Cz with reference-independent voltage mapping (average reference or PARE correction) [10] used for field topography analyses. EMG electrodes were placed on the mandible. For the TES protocol, a 0.5 Hz sine wave with 520 μA total current was delivered through two frontopolar and two inferofrontal source channels to four sink channels, located at the mastoid and back of neck (Fig. 2, right). Source/sink current alternated with the phase of the AC sine wave. To minimize sensation from the current injection, the Elefix conductive paste for the stimulating electrodes was mixed with a lidocaine solution for both stimulation and sham nights. TES was administered in five blocks of 5 min each with 1-min rest periods between blocks. The first block was started after subjects showed stable N2 sleep for 4 min. If subjects showed any signs of awakening during the TES protocol, the protocol was paused, and the remainder of the protocol was administered after the next four consecutive minutes of N2 or the start of N3. Signs of wake were determined by characterizing the streamed EEG and by video monitoring of subjects. The TES protocol was paused for two subjects. In both cases the protocol was paused and restarted twice, and all pauses occurred during a stimulation block. No TES was administered during the sham night.

### 2.4. Selection of Transcranial Electrical Stimulation targets

The selection of frontopolar and inferofrontal electrode injection sites with back of head/neck return sites was made on the basis of computational modeling of optimal stimulation sites for the limbic sources of SOs localized in our previous study (Morgan et al., unpublished work). This modeling and source localization were computed with a head conductivity model constructed for each subject. The model contained a tessellated cortical surface with 1200 dipole patches per hemisphere, each containing a dipole that had the net vector orientation of that patch’s surface. The model also contained electrode positions, determined by 3D photogrammetry [11] and registered with the MRI surface. A finite element model of the head was constructed to compute the current flow from each electrode to all cortical surface patch dipoles [12]. Source estimation of the generators of SOs from several subjects, conducted with the Bayesian Multiple Sparse Priors constraint [13] in the *Sourcerer 1.0* software (BEL, Eugene, OR, USA), indicated the primary sources for the most typical SOs lie in the medial anterior temporal area (parahippocampal gyrus) and caudal orbitofrontal limbic cortex. An example of a limbic source of a typical SO is shown in Fig. 1.

To develop TES targeting of the typical ventral anterior limbic sources (sinks), we conducted forward projections from sources in these sites in *Sourcerer* and confirmed that the optimal electrodes for TES would create source/sink inversions between inferior frontal sites (frontopolar and inferolateral frontal) and the back of the head (occipital and neck) sites, similar to the head surface voltage field pattern of the SO shown in Fig. 1b.

To contrast the current delivery montage for the present study with that used by Marshall et al. (2006) (dorsal frontal F3 & F4 sites versus mastoids), we used *Sourcerer* to estimate the current density distributed at the cortical surface for each montage (Fig. 2) [8]. Consistent with the greater (1.0 mA) current levels used by Marshall et al. (2006), this simulation suggested higher current density than with the 0.5 mA impressed current used in the present study [8]. Nonetheless, the present montage was able to deliver source/sink inversions (red-blue transitions in the palette of Fig. 2) that were well-localized at the target anterior ventral limbic sites.

### 2.5. Machine learning detection of sleep stages

In order to classify sleep stages automatically, we trained a convolutional neural network (CNN) on human scoring of 730 overnight polysomnographies (PSGs) from the Cleveland Family Study (CFS) [14–16]. Each recording was scored by a trained technician according to Rechtschaffen and Kales (R&K) criteria. We divided the PSGs into training (511), validation (110), and test (109) sets with similar age distributions. The CNN was given 30 s blocks of stacked signal data from five channels (C3-M2, C4-M1, Left EOG-M2, Right EOG-M1 and EMG) downsampled to 64 Hz. The input signals went through 10 convolutional layers (384 filters per layer), a dense layer and a softmax classification layer with five outputs for the probability of each sleep stage (Schwabedal, Hathaway, Luu, & Tucker, unpublished work). The most probable stage was taken to be the stage scored for each block. After each training epoch, the validation set was used to estimate the accuracy of the classifier and the most accurate model was stored.

**Fig. 3.**
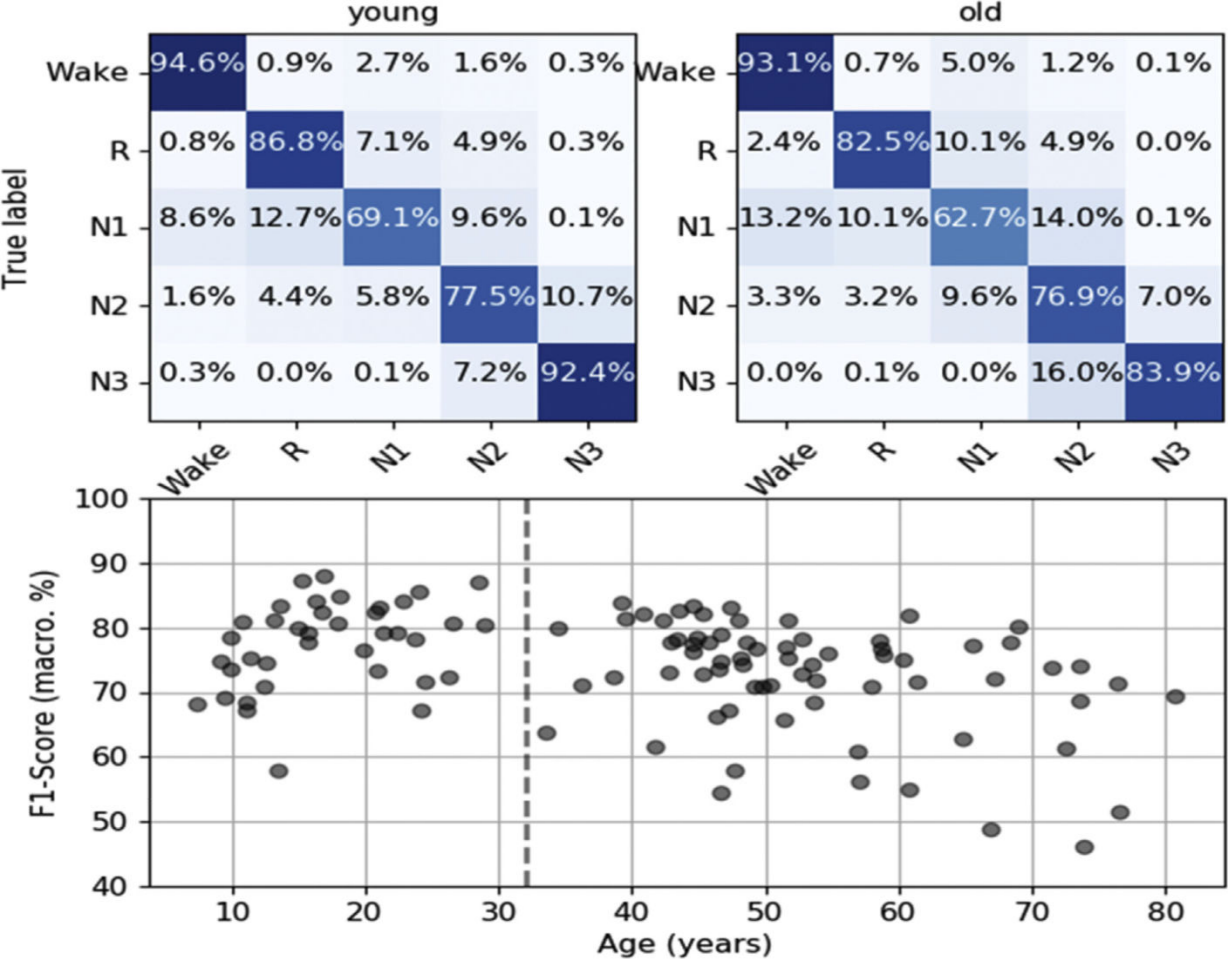
**Top:** Confusion matrices comparing the CNN sleep stage predictions to the annotations of the human scorers (true label) for young (**left**) and old (**right**) individuals. **Bottom:** Mean macro F1 score of the CNN sleep stage predictions for different ages.

The CNN predicted the human scoring of the test set with good accuracy (F1 = 73, acc = 84%, kappa = 0.77) for young and old subjects (Fig. 3). These accuracy scores fell within the range of typical interrater reliability between professional sleep scorers. For example, an analysis of two American Society for Sleep Medicine (AASM)-trained scorers’ annotations of 72 sleep recordings revealed an accuracy of 82% and kappa of 0.76 between them [17]. Another analysis of three scorers’ annotations of 21 recordings, using AASM-2007 standards, reported kappa scores of 0.80, 0.46, and 0.49 between pairs of raters [18].

The CFS data were recorded with R&K PSG positions, which place both electrooculogram (EOG) electrodes below the eyes; however, this convention has been replaced by the AASM standard, with one EOG channel above the eyes and one below the eyes. In order to demonstrate the generalizability of the ML sleep staging to the current PSG standard, we selected channels in the 256 dEEG montage that matched AASM electrode positions for our sleep recordings. We used an auxiliary electrode for EMG. Signals from these channels were fed into the CNN to make sleep stage predictions.

### 2.6. Statistical analysis

We used the ML sleep staging to calculate total time spent in each sleep stage and time spent in each stage as a percentage of total sleep time. Large artifacts introduced by TES prevented us from scoring epochs that occurred during stimulation, so these stimulation epochs were excluded from the analyses. In order to make accurate comparisons between stimulation and sham, placeholders were inserted in the sham recordings to signify when blocks of TES would have been delivered, and these segments were excluded from analysis. The 1-min rest blocks separating stimulation blocks were mostly artifact-free and the channels required for sleep staging could be used to score these epochs to examine the acute effects of stimulation. In addition to including these rest blocks in our analysis of time spent in each sleep stage throughout the whole night, we analyzed time spent in each sleep stage during the rest blocks and during the 5 min following the final stimulation block (or stimulation block placeholder for sham) in order to see if N3 increased directly following stimulation. For this particular analysis we excluded epochs following a pause in the TES protocol. With treatment (stimulation versus sham) as a within-subjects factor, a repeated-measures ANOVA was used to assess the manipulation contrast (stimulus versus sham) for each of the sleep stage duration outcome measures. A Wilcoxon signed-rank test provided an alternative nonparametric contrast for these comparisons.

As an alternative measure of slow wave (SW) activity, we analyzed spectral power in the SW (0.5–1 Hz) frequency band. For this analysis, we defined a pre-stimulation block (the 5 min preceding the first stimulation block) and a post-stimulation block (TES rest blocks, plus 5 min following the final stimulation block). For each block of interest, segments of clean data lasting at least 5 s were marked by hand. The 20 s of data following each stimulation block (and equivalent segments for sham) were automatically excluded because of TES-induced artifact. A Wilcoxon signed-rank test revealed that the total duration of clean data did not differ between stimulation (*M* = 522.59 s) and sham (*M* = 551.55 s) conditions (*T* = 17.0, *p* = 0.944). Clean data segments were tapered with a Tukey window (α = 0.2) and then concatenated separately for pre-stimulation and post-stimulation. Periodograms were computed for the concatenated signals using Welch’s method with a Hanning window (2.56-sec segments and 1.28-sec overlap between segments). Absolute PSD in the SW frequency band was calculated by averaging the PSD values for all frequency bins contained in the 0.5–1 Hz band for post-stimulation blocks. Relative PSD in the SW frequency band was defined as the percent increase in SW band PSD between pre-stimulation and post-stimulation. We analyzed these measures of absolute and relative PSD in 10 different scalp regions by averaging PSD in clusters of channels comprising each region. We excluded one outlier who was awake during the majority of the TES protocol and we excluded another subject because of a lack of clean segments post-stimulation. This left us with eight subjects for the spectral analysis. Because the data were not normally distributed, a Wilcoxon signed-rank test was used to compare absolute and relative SW band PSD between stimulation and sham for each scalp region.

## 3. Results

**Fig. 4.**
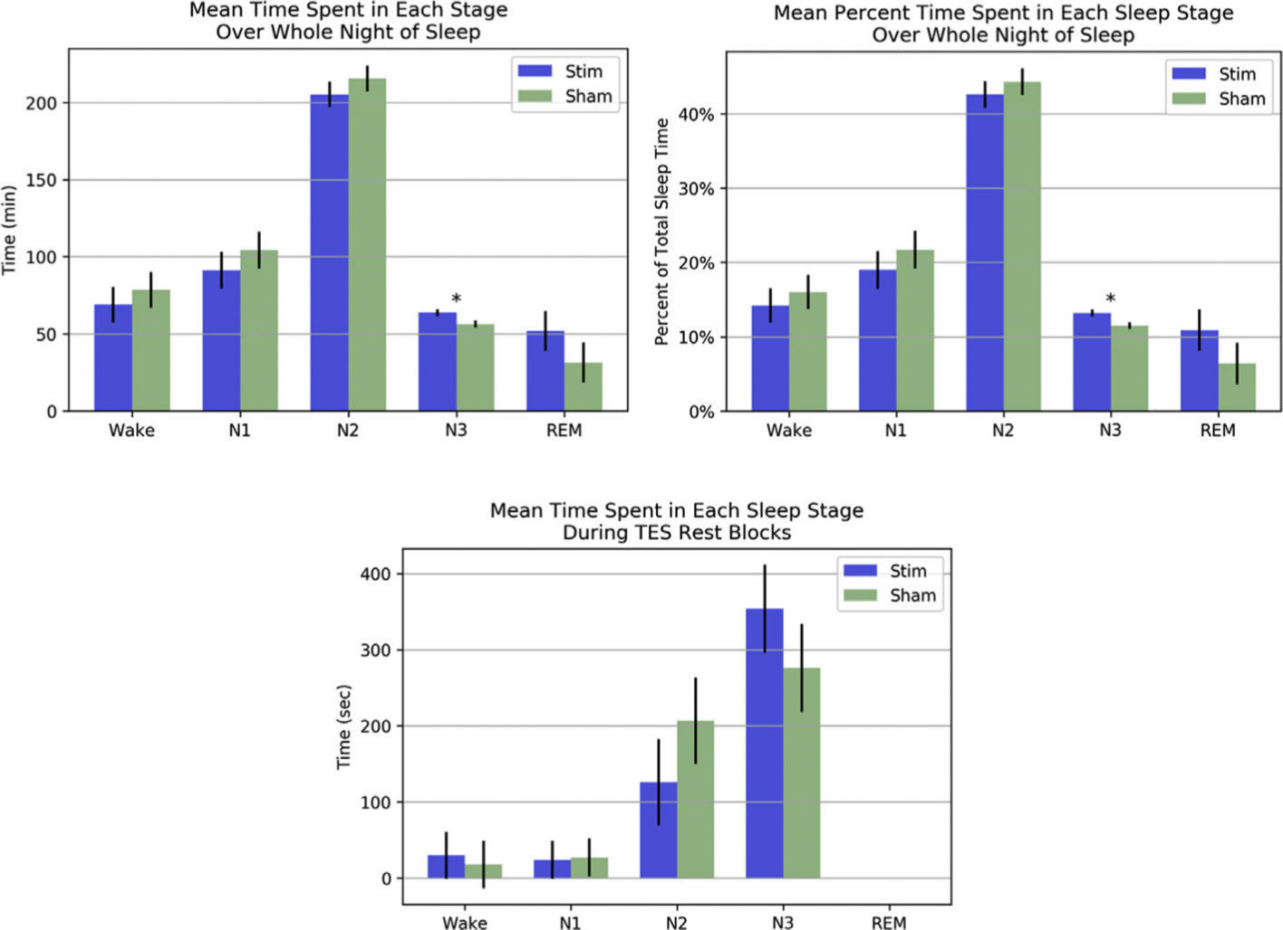
Mean time spent in each sleep stage for stimulation and sham conditions. **Top left:** Raw time (minutes) spent in each sleep stage over the whole night. **Top right:** Time spent in each sleep stage as a percentage of total sleep time. **Bottom:** Time (seconds) spent in each sleep stage during 1-min TES rest blocks and 5 min following the final stimulation block. Error bars represent standard error of the difference scores between stimulation and sham conditions. An asterisk indicates a significant difference (*p < 0.05).

The sham (placebo) control was effective; subjects were unable to guess beyond chance on which night the stimulation was administered. As hypothesized, the repeated measures ANOVA revealed that subjects spent more time in N3 during the stimulation night (*M* = 63.75 min) than during the sham night (*M* = 56.35 min; *F* [1,9] = 9.06, *p* = 0.015 for the ANOVA and *T* = 5.0, *p* = 0.025 for the one-tailed Wilcoxon signed-rank). There was a greater percentage of time in N3 for stimulation (*M* = 13.2%) versus sham (*M* = 11.5%; *F* [1,9] = 10.76, *p* = 0.010 for the ANOVA and *T* = 5.0 *p* = 0.025 for the one-tailed Wilcoxon signed-rank). Neither time in minutes nor percent time spent in other sleep stages differed significantly between stimulation and sham conditions (means and standard errors are presented in Fig. 4, top).

We found considerable individual differences in N3 duration, and these were related to the age of the subjects, with older subjects having less N3. The Pearson’s correlation between age and N3 duration for the stimulation condition was (*r* [9] = −0.56, *p* = 0.09). Similar negative correlations with age were observed for N3 duration in the sham condition and for the percent N3 measures in stimulation and sham conditions (*r* = −0.59, −0.56, and −0.56, respectively). Although N3 thus clearly declines with age even in this healthy sample, the difference in N3 duration between stimulation and sham did not correlate significantly with age (*r* [9] = −0.29, *p* = 0.41) (see Table 1).

Examining the immediate effects of stimulation on neural activity of sleep, we conducted analyses on the TES rest blocks and 5 min following the final stimulation block. In this window subjects typically remained in stages N2 and N3 (Fig. 4, bottom). Excluding one outlier who was awake during most of these epochs on the stimulation night, there was no significant difference in time spent in N2 (*F* [1,8] = 0.68, *p* = 0.435 for the ANOVA and *T* = 11.0, *p* = 0.360 for the two-tailed Wilcoxon signed-rank) or time spent in N3 (*F* [1,8] = 2.01, *p* = 0.194 for the ANOVA and *T* = 7.0, *p* = 0.070 for the one-tailed Wilcoxon signed-rank) between stimulation and sham in this immediate window. Although these differences were not significant, there was an observable trend in the data such that subjects spent more time in N3 on the stimulation night (*M* = 354 s) than on the sham night (*M* = 276 s) and more time in N2 on the sham night (*M* = 207 s) than on the stimulation night (*M* = 126 s). We present time spent in each sleep stage directly following stimulation blocks for each subject and condition in Table 2. As demonstrated in Table 2, one subject severely broke the trend, presenting N2 for a large majority of epochs during the stimulation night and N3 for a large majority of epochs during the sham night. This drove up the variance of the difference scores such that these large mean differences in N2 and N3 duration following stimulation blocks were not significant.

**Fig. 5.**
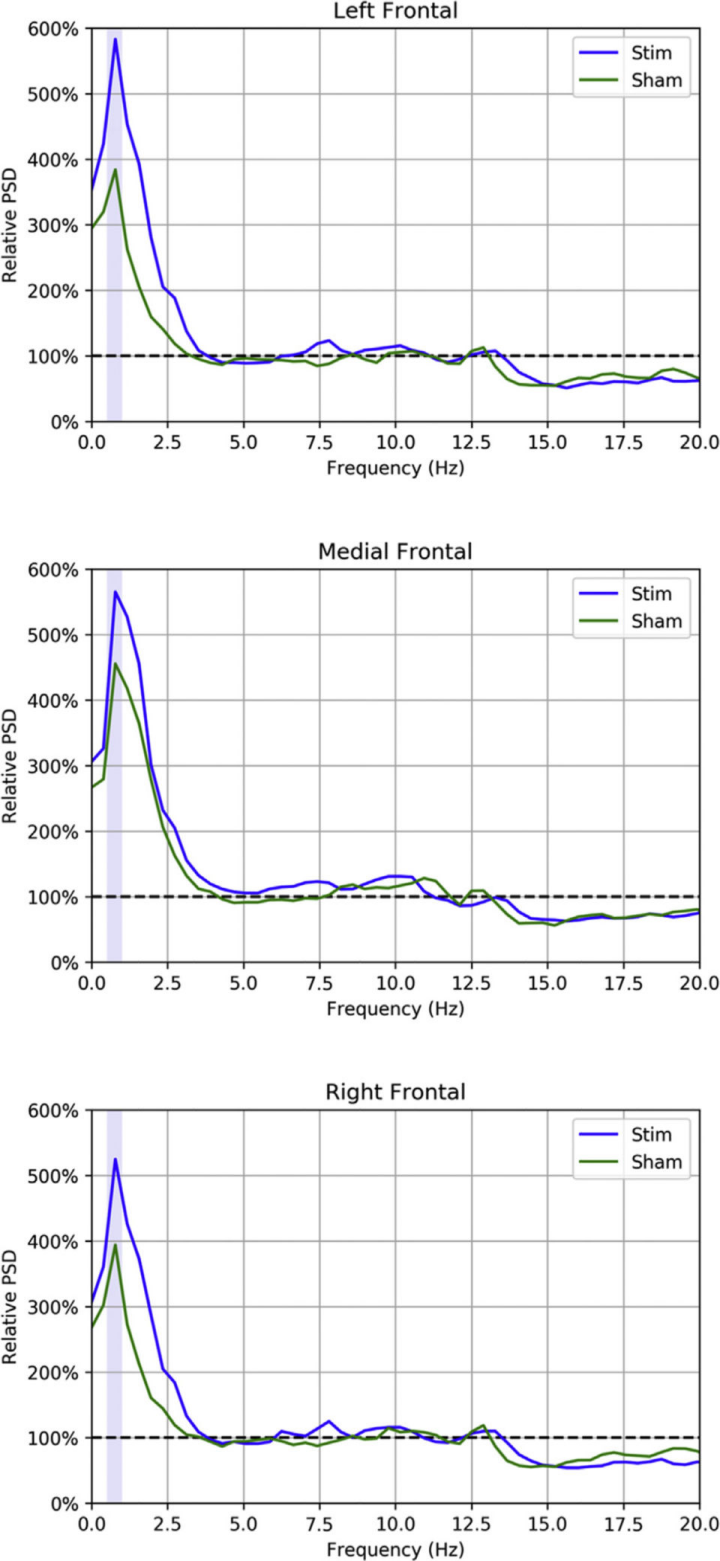
Post-stimulation PSD relative to pre-stimulation PSD for left frontal (**top**), medial frontal (**middle**), and right frontal (**bottom**) scalp regions. Data are averaged across subjects. The shaded area shows the 0.5–1 Hz frequency band of interest.

For each treatment condition and scalp region, we report mean absolute (Table 3) and relative (Table 4) PSD in the SW frequency band. As demonstrated in Table 3, there were no significant differences in absolute post-stimulation SW band power between stim and sham. However, the relative PSD results are more telling. Table 4 shows that the largest increase in SW band power across treatment conditions was in the medial prefrontal region (*M* = 543.7%). The increase in SW band power depended on the laterality of the scalp region for the stimulation condition, but not for sham. A two-tailed Wilcoxon signed-rank test revealed that for the stimulation condition, the increase in SW band power was greater in the left temporoparietal region (*M* = 626.6%) than in the right temporoparietal region (*M* = 538.0%; *T* = 1.0, *p* = 0.021), but for sham, there was no difference between these regions (*T* = 14.0, *p* = 0.624). The one-tailed Wilcoxon signed-rank tests comparing stimulation and sham indicated there was a greater percent increase in SW band power for stimulation than for sham in the left temporoparietal (*T* = 3.0, *p* = 0.021) and left occipital (*T* = 5.0, *p* = 0.040) regions, but no significant difference between stimulation and sham for other regions. This left-lateralization of the increase due to TES synchronization of SOs is consistent with the left-lateralization of SOs observed in the source localization results of Morgan et al. (unpublished). A closer look at the means in Table 3 reveals that the percent increase in SW band power in medial prefrontal and medial frontal regions is comparable between stimulation and sham, whereas for the lateral frontal regions, the difference between stimulation and sham is more pronounced. This phenomenon can also be observed in Fig. 5, where we plot percent change in PSD by frequency for left frontal, medial frontal, and right frontal regions. Here we see that although the largest increases in SW band power are in the medial frontal region, the largest differences between stimulation and sham are observed on the left and right.

## 4. Discussion

In light of the recent controversy over whether TES, as commonly implemented, can influence neural activity with small currents applied at the head surface [19], the present results show that even small currents (~0.5 mA) are able to synchronize brain oscillations when applied with appropriate anatomical targeting and neurophysiological timing. The spatial targeting of TES in the present study included computational modeling (Fig. 2) to place electrodes to optimally target the limbic sources of slow oscillations (SOs). The timing of TES application was delayed until after a period of N2 sleep, when the brain normally transitions to N3. As expected, the slow oscillatory TES was effective in synchronizing N3 SOs immediately, as shown by the enhanced N3 duration during the rest blocks and immediately after stimulation. This finding is strengthened by the significantly increased 0.5–1 Hz band power in left temporoparietal and left occipital regions following TES. Furthermore, the fact that the total N3 duration for the night was significantly enhanced by TES, leading to 13% longer N3 sleep compared to the sham control, suggests that the synchronization by externally applied currents was sufficient to entrain the SOs of deep sleep that were then maintained as endogenous neurophysiological activity beyond the TES windows.

Unexpectedly, the TES synchronization of SOs in the first N3 period led to greater REM sleep later in the night. Whereas a decrease in other sleep stages would be expected as a result of increasing the duration of N3 (if total sleep time remained constant), this increase of REM (although not significant in this small sample) may suggest a global change in sleep architecture and should be examined in future studies.

Further research on external electrical synchronization of SOs of deep sleep may have both practical and theoretical significance. Practically, applying TES for an immediate enhancement of N3 sleep has been shown to improve daytime memory performance in both young and older adults [8,9]. Older persons with Mild Cognitive Impairment (MCI) have considerably poorer sleep than healthy seniors [20]. Given the significant decline in memory experienced by many older adults, a simple procedure for maintaining strong deep sleep beyond age 30 may be a useful approach to successful aging.

Furthermore, several findings suggest that the decline of deep sleep may be a causative factor in the progression to dementia. Measures of amyloid plaque accumulation, thought to lead to excitotoxic damage of neural tissue in Alzheimer’s Disease, do not predict memory decline in older subjects unless those subjects also exhibit a decline in N3 sleep [1]. A mechanism for this apparent protective role of deep sleep in avoiding dementia has been suggested by the recent observation that the physiologic activity of SOs changes brain volume with each SO event. During this volumetric change, sufficient hydrodynamic force moves CSF out of the cranial volume thereby facilitating the lymphatic excretion of amyloid and tau neurotoxins [4]. If a simple procedure for TES synchronization of SOs can be made practical for routine use, it may enhance the health benefits of deep sleep and decrease the risk for dementia that accrues with increasing age.

Theoretical insight into the mechanism through which SOs organize brain activity may be gained through manipulation of TES synchronization in relation to specific memory consolidation tasks. An interesting question is how the large (75 μV) limbic field fluctuations associated with SOs may regulate limbic-neocortical consolidation dynamics [21] in response to the synchronizing effects of claustrum projections [22,23]. In preliminary observations, we have noted that some small sources in neocortex, coupled in time with the large limbic sources, invert in phase with the large limbic sources (in the phase transition from the frontal-negative SO peak to the small frontal-positive following wave, as shown in Fig. 1a & d). Source modeling shows that the EEG recordings of most SOs are fully explained by the limbic sources; the associated neocortical sources are invariably small and contribute negligibly to the SO features observed in the EEG (Morgan et al., unpublished work). Another question remaining is whether our use of 4 source electrodes (whereas other studies used 2) led to wider current spread, despite the weaker current level used in the present study (0.5 mA in ours vs 1 or 2 mA in previous studies). Future research should include a more accurate model of current flow using physical models of each individual’s brain and head geometry.

Nonetheless, the small phase-aligned neocortical sources are measurable in many SO samples, and they should be separable in theory from the limbic electric fields in source analysis with dense array EEG. Intracranial recordings in human epileptic patients have shown that the down states associated with SOs are often observed in local neocortical sites [24]. Furthermore, a typical pattern in the intracranial recordings are down states in medial frontal lobe that propagate to medial temporal sites [24], perhaps consistent with the noninvasive source localization results of Morgan et al. In future noninvasive studies of sleep and memory, analysis of the source waveforms from both limbic and neocortical sites, with interactions inferred with appropriate nerve conduction times for cortico-cortical connections, would be instructive to combine with experimental manipulation of anatomically targeted memory consolidation tasks.

The left-lateralization of the SO sources in the Morgan et al. (unpublished) study was observed for posterior non-limbic neocortical sites, including the fusiform gyrus. In the present results, left-lateralization was observed for the slow wave power increases as a result of TES in both temporal and occipital areas. The implication may be that the response to treatment is greatest in areas with the largest endogenous generation of SOs. Why these SO generators are stronger in the human left hemisphere is an interesting question for future research.

Although functional interpretation must await more analytic experiments, the noninvasive EEG source localization results indicate that focal regions of limbic cortex, and not global waves over lateral neocortex, create the large potentials of SOs observed in the EEG during human deep sleep. For such focal limbic events to create the impressively large fields of SOs in the EEG implies that these events must involve highly synchronous local generators. By understanding the ventral limbic location of these synchronous SO generators, the present results suggest that targeting them specifically with artificial synchronizing currents can enhance the SO activity to extend the duration, and perhaps the health benefits, of deep sleep.

## Figures and Tables

**Table 1:** Total time spent in N3 (min) and percent time relative to total sleep time for each subject and condition.

Subject	N3 Total Time (min)			N3 Percent Time		
	Stimulation	Sham	Difference	Stimulation	Sham	Difference
1	20.0	13.5	6.5	4.3%	3.0%	1.9%
2	64.0	56.5	7.5	13.6%	11.7%	2.7%
3	130.5	117.5	13.0	26.1%	23.4%	4.5%
4	80.5	57.5	23.0	16.4%	11.9%	1.6%
5	31.0	24.5	6.5	7.1%	5.5%	2.9%
6	134.5	121.5	13.0	27.7%	24.8%	0.1%
7	12.0	11.5	0.5	2.3%	2.2%	−0.5%
8	38.0	41.0	−3.0	7.5%	8.0%	−0.4%
9	65.0	66.5	−1.5	13.7%	14.1%	2.4%
10	62.0	53.5	8.5	13.1%	10.7%	1.7%
Mean	63.75	56.35	7.40	13.2%	11.5%	1.7%
SD	42.26	38.29	7.77	8.5%	7.7%	1.6%

**Table 2:** Time (seconds) spent in each sleep stage during 1-min TES rest blocks and 5 min following the final stimulation block.

Subject	Wake		N1		N2		N3		REM	
	Stim	Sham	Stim	Sham	Stim	Sham	Stim	Sham	Stim	Sham
1	300	0	210	0	30	390	0	90	0	0
2	0	0	0	0	0	300	540	240	0	0
3	0	0	0	0	0	0	540	480	0	0
4	0	0	0	30	120	150	420	360	0	0
5	0	60	0	150	60	240	480	90	0	0
6	0	0	0	30	60	240	480	270	0	0
7	0	0	0	0	90	180	450	360	0	0
8	0	0	0	0	120	210	420	330	0	0
9	0	0	0	0	450	120	90	420	0	0
10	0	120	30	60	330	240	120	120	0	0
Mean	30	18	24	27	126	207	354	276	0	0

**Table 3:** Means table for absolute PSD (μV^2^/Hz) in the SW band (0.5–1 Hz) during the TES rest blocks and 5 min following the final stimulation block. In the far-right column we report the p-values for the one-tailed Wilcoxon signed-rank tests comparing stimulation and sham.

	Stim	Sham	Mean	Wilcoxon p-value
Left Frontal	721.47	656.07	688.77	0.264
Right Frontal	680.48	631.04	655.76	0.363
Medial Prefrontal	650.08	596.68	623.38	0.472
Medial Frontal	175.81	161.52	168.66	0.583
Left Temporoparietal	276.19	230.48	253.34	0.264
Right Temporoparietal	272.47	247.08	259.77	0.312
Posterior Parietal	185.25	188.24	186.74	0.472
Left Occipital	659.14	629.47	644.30	0.312
Right Occipital	677.05	646.54	661.80	0.363
Medial Occipital	737.23	713.75	725.49	0.363
Mean	503.52	470.09		

**Table 4:** Means table for percent increase in SW band (0.5–1 Hz) PSD between pre-stimulation and post-stimulation. In the far-right column, we report the p-values for the one-tailed Wilcoxon signed-rank tests comparing stimulation and sham.

	Stim	Sham	Mean	Wilcoxon p-value
Left Frontal	583.2%	384.1%	483.6%	0.054
Right Frontal	524.8%	394.3%	459.5%	0.092
Medial Prefrontal	589.6%	497.8%	543.7%	0.264
Medial Frontal	565.3%	455.6%	510.4%	0.147
Left Temporoparietal	626.6%	383.7%	505.2%	0.021*
Right Temporoparietal	538.0%	375.3%	456.6%	0.071
Posterior Parietal	458.4%	335.9%	397.1%	0.054
Left Occipital	528.7%	352.3%	440.5%	0.040*
Right Occipital	508.3%	361.9%	435.1%	0.092
Medial Occipital	493.3%	345.4%	419.3%	0.092
Mean	541.6%	388.6%		

An asterisk indicates a significant difference (*p < 0.05).
